# Opinion Dynamics With Mobile Agents: Contrarian Effects by Spatial Correlations

**DOI:** 10.3389/frobt.2018.00063

**Published:** 2018-06-06

**Authors:** Heiko Hamann

**Affiliations:** Institute of Computer Engineering, University of Lübeck, Lübeck, Germany

**Keywords:** swarm robotics, swarm intelligence, opinion dynamics, collective decision making, swarm robotic system

## Abstract

We investigate the dynamics of opinion formation in a group of mobile agents with noisy perceptions. Two models are applied, the 2-state Galam opinion dynamics model with contrarians and an urn model of collective decision-making. It is shown that models built on the well-mixed assumption fail to represent the dynamics of a simple scenario. The challenge of accounting for correlations in the agents' spatial distribution is overcome by different heuristics and supported by empirical investigations. We present a concise, simple 1-dimensional macroscopic modeling approach that can be tuned to correctly model spatial correlations.

## 1. Introduction

Group behaviors of interacting, mobile agents are of interest in many fields and many models have been published. So-called microscopic models (also known as multi-agent models, agent-based models, or individual-based models) explicitly incorporate properties of each member of the group such as position, direction, and internal state. Examples are models of self-propelled particles (Vicsek et al., 1995; Czirók and Vicsek, 2000; Levine et al., 2000) and active Brownian agents (Schimansky-Geier et al., 1995; Helbing et al., 1997; Schweitzer, 2003). So-called macroscopic models abstract away such individual properties (e.g., derivations in the mean-field limit) and reduce the state space to a few variables. Examples are diffusion models of animal groups (Okubo, 1986; Hillen and Painter, 2009; Degond and Yang, 2010; Vicsek and Zafeiris, 2012), robots (Galstyan et al., 2005; Hamann, 2010, 2018; Prorok et al., 2011), and general models of self-propelled particles (Czirók and Vicsek, 2000). Collective decision-making, in particular, is observed in many systems such as natural swarms (Franks et al., 2003; Nicolis and Dussutour, 2008; Yates et al., 2009), artificial swarms (Schmickl et al., 2008; Garnier et al., 2009), and in human groups and societies (Galam and Moscovici, 1991; Helbing and Molnar, 1997; Hegselmann and Krause, 2002; Galam, 2004; Galam and Jacobs, 2007; Motsch and Tadmor, 2014). Naturally, observations and descriptions of these systems take place on two different levels: the microscopic level, where an individual agent is observed and described, and the macroscopic level, where the group of agents is considered as a whole. This categorization holds also for models of opinion dynamics. Microscopic models represent internal states and in the case of spatial models also positions of each agent which increases the computational effort that is to be invested to evaluate the model. In macroscopic models one abstracts from details of individual agents, for example, in a mean-field approach (Schweitzer, 2002), and tries to focus on important macroscopic features. The macroscopic models are the epistemologically more promising approach because they allow for deeper insights as stated by Schweitzer (2003):

“To gain insight into the interplay between microscopic interactions and macroscopic features, it is important to find a level of description that, on the one hand, considers specific features of the system and is suitable for reflecting the origination of new qualities, but, on the other hand, is not flooded with microscopic details.”

There are macroscopic models that are built on simplifying assumptions, for example, there are models of opinion dynamics that assume well-mixed agent distributions (Schweitzer et al., 2002; Galam, 2004), that is, uniform distributions of agents independent of their current opinion. While it is possible, for example, to derive a Fokker-Planck equation of Brownian motion with drift based on integration over short time intervals assuming uncorrelated collisions of particles (Haken, 1977), it is in general not possible for biological swarm models due to the breakdown of the “propagation of chaos” (Carlen et al., 2013).

A frequently used method to incorporate spatial correlations of agents and interactions (Mateo et al., 2017), be it due to spatial relations or relations based on opinions, is that of voter models based on networks. Opinion dynamics models and swarm models have both two different types: discrete (Sood and Redner, 2005; Holme and Newman, 2006) and continuous (Toner and Tu, 1998). Whether spatiality is of importance in swarm and opinion dynamics models is questioned. For example Huepe et al. (2011) argue that

“spatial geometry may have less of an impact on collective motion than previously thought.”

A simple modeling approach is based on so-called “adaptive coevolutionary networks” which are of low dimension and non-spatial (Gross and Blasius, 2008; Huepe et al., 2011).

We consider the well-mixed assumption as too imprecise for certain applications (Hamann, 2012, 2013) because agent distributions might be intrinsically correlated and consequently models based on the well-mixed assumption have limited accuracy. These applications, such as collective motion of locusts (Yates et al., 2009), hung elections (Galam, 2004), or aggregation behaviors of robot swarms (Schmickl and Hamann, 2011), are of importance. Hence, we assume that spatial correlations exist but we also want to restrict ourselves to very concise and easy to handle models of low dimensions. The motivation of this paper is to show how the limitations of the well-mixed assumption can be overcome while still keeping the models concise and easily manageable.

In the following, we investigate a binary decision problem in a group of mobile agents with noisy perceptions and compare results of two opinion dynamics models: first, the 2-state Galam opinion dynamics model with contrarians (Galam, 2004) and, second, an urn model for collective decisions in swarms (Hamann, 2012). The Galam model is particularly suited to the investigated multi-agent system because it accounts for the size of subgroups, that influence each others' opinion, which is also explicitly set in the multi-agent system. However, it does not account for spatial correlations between agents. The urn model is of interest because it allows for a description of spatial correlations but has no concept of subgroup sizes.

The multi-agent system that is investigated here was introduced before (Hamann and Wörn, 2007; Hamann et al., 2010) and was labeled “density classification scenario” because the agents' choice is set close to a symmetric setting initially and the supposed task is that all agents should converge to the choice that had a slight majority initially. Here, we are not interested in collective decision-making as such but only the spatial correlations of the opinion dynamics. The agents show a simple form of motion. They move like billiard balls without friction. They move straight within a square and bounce off each other and the bounding walls.

## 2. Density classification scenario

In this scenario, we have a population of *N* agents that are in one of two states: either they are in favor of opinion *A* or in favor of opinion *B*. Originally this scenario is interpreted as a task that is assigned to a population to estimate whether there are initially more *A*- or more *B*-members, that is, to converge on a majority decision (Hamann and Wörn, 2007; Hamann et al., 2010). This problem is derived from a well-known example of emergent computation in cellular automata (Packard, 1988).

We define this system as a 2-d self-propelled particles model. The particles move in a bounded square of dimensionless side length 1 (unit square). Collisions between particles and bounds are elastic.

Paricles also avoid collisions with each other by bouncing off as soon as they are within a collision avoidance radius *r* = 0.01.^1^ All particles have equal velocity of 0.01 at all times (see Table 1 for all parameters). Particle positions **x**(*t*) and states *o*(*t*) have initially a random uniform distribution (i.e., initial positions sampled from a uniform distribution; 50% of agents in favor of *A*, 50% in favor of *B*).

**Table 1:** Parameters of the density classification scenario and the values used for simulations.

**Parameter**	**Value**
Swarm size *N*	150
Square world side length	1
Avoidance distance	0.01
Agent speed per step	0.01
Iterations per simulation run	8 × 10^3^
Repetitions *M* (independent sim. runs)	1 × 10^6^

We include an explicit stochastic component because we assume errors in the opinion recognition. We assume that a particle recognizes the state of an encountered particle correctly only with a given probability 1−γ = 0.8. A particle perceives the state of particle *j* as

(1) $$p(o_{j}(t))=\{\begin{array}{ll} o_{j}(t), & \text{ with probability }1-\gamma \\ \bar{o_{j}}(t), & \text{ with probability }\gamma \end{array},$$

whereas $\bar{o_{j}}$ is the opposite of the opinion of particle *j*.

The particles have an internal memory N. Whenever at least two particles *i* and *j* are mutually within perception range *r* = 0.01 (||**x**_*j*_(*t*)−**x**_*i*_(*t*)|| ≤ *r*), they perceive the opinion of each other (*p*(*o*_*i*_(*t*)) and *p*(*o*_*j*_(*t*)) respectively), and store it in their memory $N_{i}\cap \left\{p\left(o_{j}\left(t\right)\right)\right\}$ an $N_{j}\cap \left\{p\left(o_{i}\left(t\right)\right)\right\}$ respectively. Once a particle had |N|=5 of these particle–particle encounters^2^, it reconsiders its current opinion, converts to the opinion that was more frequent in these five encounters, and resets its memory to N=∅. The above given parameters are set as stated in Table 1, such that a particle does not travel far (i.e., only fractions of the unit square) to gather five opinions. Hence, the system is not necessarily well-mixed, there is a chance for spatial correlations to form, and a particle's memory N can be interpreted as its perception of its neighborhood.

## 3. Modeling approach i: galam model

In the following we apply the 2-state Galam opinion dynamics model (Galam and Moscovici, 1991; Galam, 1997, 2008). It is a non-spatial model with discrete time and based on a population of *N* agents. In each round, agents come together in small groups of size *m* that are randomly picked without any bias. Within these groups a local majority rule is applied (i.e., the whole group switches to the group's majority opinion). If *m* is odd, tie breakers need not to be considered.

The density classification task is similar to the 2-state Galam opinion dynamics model concerning the decision process which is based on observing five particles and subsequently switching to the state of the majority. However, the formation of these virtual groups is neither necessarily mutual due to asynchronous decisions nor uncorrelated due to the spatial distribution of particles. Still, we apply Galam's model as an approximation. We set the group size to *m* = 5. In addition we apply Galam's extension of his model, the so-called “contrarians” (Galam, 2004). Galam's assumption is that a fraction *a* of the population are contrarians, that is, they always switch to the minority opinion of their group. We use the contrarian concept here to model effects due to spatial correlations, which will become clear in the following.

The model is based on one state variable *s*_*t*_. Say we count a number of *A*_*t*_ agents with opinion *A* at time *t*, then we define the global opinion state *s*_*t*_ = *A*_*t*_/*N* which is the fraction of the population with opinion *A*. The dynamics of the state variable *s*_*t*_ according to the 2-state Galam opinion dynamics model with contrarians for a group size of *m* = 5 is

(2) $$\begin{array}{l} s_{t+1}=g(s_{t},a)=(1-a)(10s_{t}^{3}{\left(1-s_{t}\right)}^{2}+5s_{t}^{4}(1-s_{t})+s_{t}^{5})\\ \;+a(10{\left(1-s_{t}\right)}^{3}{\left(1-(1-s_{t})\right)}^{2}+5{\left(1-s_{t}\right)}^{4}\left(1-(1-s_{t})\right)\\ \;+{\left(1-s_{t}\right)}^{5}). \end{array}$$

**Figure 1:**
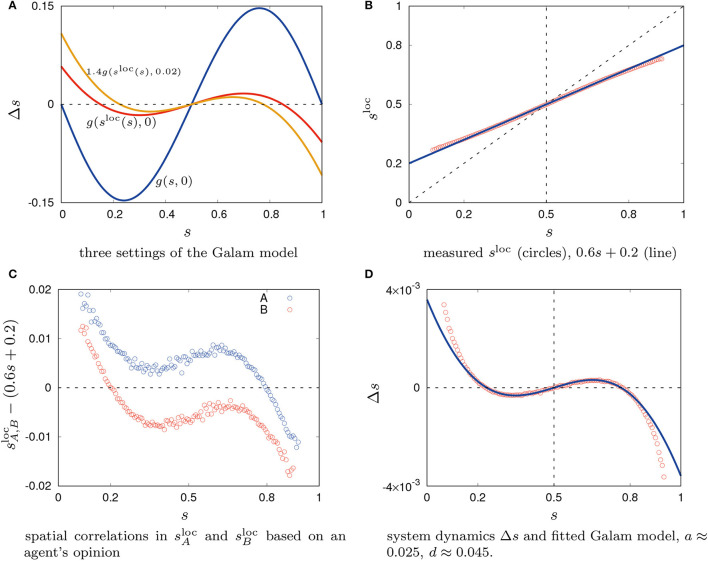
System dynamics Δ*s* for the Galam model; measurements of spatial correlations and Δ*s* in the agent-based model.

This model is based on simple probability theory and combinatorics, for details see Galam (2004). In Figure 1A we give a plot for Δ*s*_*t*_ = *s*_*t*+1_−*s*_*t*_ = *g*(*s*_*t*_, *a*)−*s*_*t*_ with *a* = 0. For *g*(*s*_*t*_, *a* = 0) we have two stable fixed points ($s_{1}^{*}=0$ and $s_{2}^{*}=1$)^3^. Due to the noisy perception of particles in the density classification task, this does not well correspond to the observation in the simulations. Even for *s* = 0 agents will on average still perceive an effective state of *s* = γ (discussed below concerning Figure 1B).

Next, we want to empirically investigate the spatial correlations between particles in the density classification simulation. We define the local perception $s_{i}^{\text{loc}}\left(s,t\right)$ of the global state *s* by a particle *i* as

(3) $$s_{i}^{\text{loc}}(s,t)=\{\begin{array}{ll} \frac{1}{\left|N_{i}(t)\right|}|\{o|o=A,o\in N_{i}(t)\}|, & |N_{i}(t)|>0\\ \text{undefined}, & |N_{i}(t)|=0 \end{array}.$$

In order to get statistically useful measurements, we define the local opinion state *s*^loc^(*s, t*) as the average over an ensemble of *M* independent simulation runs and over a population *N* for a given global opinion state *s*

(4) $$\begin{array}{r} s^{\text{loc}}\left(s,t\right)=\frac{1}{MN}\underset{M}{\sum }\underset{N}{\sum }s_{i}^{\text{loc}}\left(s,t\right). \end{array}$$

*s*^loc^(*s, t*) is the state of the neighborhood as it is perceived locally by an agent of any opinion including the particles' noisy perceptions and averaged over an ensemble of simulation runs. The relation between *s*^loc^(*s, t*) and *s*(*t*) was determined empirically and as expected it was found to be almost linear, see Figure 1B. Hence, we follow a two-step process of first accounting for the known influence by the agent's imperfect perception (γ) and then studying the remaining deviation. We approximate it linearly

(5) $$\begin{array}{r} s^{\text{loc}}\left(s,t\right)=c_{1}s\left(t\right)+c_{2}. \end{array}$$

For perfect perception γ = 0 we would expect $s^{\text{loc}}\left(s,t\right)\approx c_{1}s\left(t\right)$. Here we have γ = 0.2. *s*^loc^ is time-variant but converges approximately to

(6) $$\begin{array}{r} s^{\text{loc}}\left(s,t^{\prime }\right)\approx \left(1-2\gamma \right)s\left(t^{\prime }\right)+\gamma , \end{array}$$

for *t*′≫0. This is a modeling approach based on a well-mixed assumption, not an attempt to fit the measured data. In a well-mixed system (without any spatial correlations) an agent perceives a local state of *s*^loc^ = γ for *s* = 0 and *s*^loc^ = 1−γ for *s* = 1. For γ = 0.2 we get $s^{\text{loc}}\left(s_{t}^{\prime }\right)\approx 0.6s_{t^{\prime }}+0.2$ (plotted in Figure 1B). A plot of $g\left(s^{\text{loc}}\left(s_{t}\right),0\right)$ in Figure 1A shows the effect of *s*^loc^. The two stable fixed points move inwards toward the middle and the absolute values decrease.

$g\left(s^{\text{loc}}\left(s_{t}\right),a=0\right)$ gives the exact values of Δ*s*_*t*_ for an assumed well-mixed setting, that is, if an agent's current opinion is truly uncorrelated with its position. However, the spatial distribution of agents is not independent from the agents' opinion. A spatial correlation is likely to emerge based on the definition of the agent's behavior. The opinion of an agent is directly influenced by its neighbors, consequently they are correlated. This can also be determined in simulation by measuring the average, locally perceived opinion state for agents of a given opinion (measured during the last 100 time steps of the simulation, that is, 7, 900 < *t* ≤ 8, 000); $s_{A}^{\text{loc}}\left(s\right)$ gives the state perceived by agents of opinion *A* and $s_{B}^{\text{loc}}\left(s\right)$ gives the state perceived by agents of opinion *B*. The differences between $s_{A,B}^{\text{loc}}\left(s\right)$ and 0.6*s*+0.2 show clearly a bias depending on the agents' current opinion as seen in Figure 1C which is evidence of a correlated spatial distribution of agents. The neighborhood of an agent with opinion *A* is populated by more agents of opinion *A* than on average for any state (for *s* < 0.8) and respectively for agents with opinion *B*.

We need an adjustment of the Galam model to account for spatial correlations. Although there is no explicit concept of contrarians in the density classification scenario, the contrarian approach can be used to compensate the effect of spatial correlations. That way the contrarians reflect the observed bias away from the current global majority due to local effects. For s < 0.5 contrarians model the excess of perceived particles with opinion o = A by particles with opinion *A* and for *s*>0.5 contrarians model the excess of perceived particles with opinion o = B by particles with opinion *B*. With increasing contrarian density *a* the two stable fixed points move further inwards until they would unite for *a*≈0.0555 leaving one stable fixed point at *s* = 0.5. In addition, we also scale the absolute values of $g\left(s^{\text{loc}}\left(s_{t}\right),a\right)$ by multiplying a constant *d* as done in Figure 1A. Fitting $dg\left(s^{\text{loc}}\left(s_{t}\right),a\right)$ via *a* and *d* to the empirically obtained data gives a good result for values 0.17 < s < 0.83 but has systematic errors outside of that interval, see Figure 1D^4^. Data was obtained by measuring values of Δ*s*_*t*_ as a function of the current system state *s*_*t*_ (i.e., Δ*s*_*t*_(*s*_*t*_) = *s*_*t*+1_−*s*_*t*_) during the last 100 time steps of the simulation, that is, 7, 900 < *t* ≤ 8, 000. Plotted values are averages over all samples collected of the respective Δ*s*_*t*_(*s*_*t*_).

## 4. Modeling approach ii: urn model

As an alternative to Galam's model we apply an urn model that was originally introduced as a model of collective decision-making in swarms (Hamann, 2012). The main idea is that we have a state-dependent probability of positive feedback *P*_fb_(*s*). The current majority opinion spreads for *P*_fb_(*s*)>0.5 and is diminished otherwise.

The idea of this urn model is as follows. An urn is filled with *N* agents which are either associated with opinion *A* or *B*. The game's dynamics is turn-based. First an agent is drawn with replacement and its opinion is noted. Then the opinion of a second agent is changed determined by that noted opinion. Say, first, an agent with opinion *A* is drawn. The probability of drawing an agent with opinion *A* is implicitly determined by the current number of agents with opinion *A* in the urn. The subsequent change of opinion of a second agent is determined by the probability of positive feedback *P*_fb_(*s*) and effects either a positive (an agent in the urn changes from opinion *B* to *A*, the fraction of the first drawn agent increases) or a negative feedback (an agent changes from opinion *A* to *B*, the fraction of the first drawn agent decreases). The feedback is determined explicitly by probability *P*_fb_(*s*) that we define below and that also depends on the current global opinion state *s*. Following Hamann (2012), the state variable's dynamics is defined by^5^

(7) $$\begin{array}{r} \Delta s_{t}=s_{t+1}-s_{t}=4e\left(P_{\text{fb}}\left(s_{t}\right)-0.5\right)\left(s_{t}-0.5\right), \end{array}$$

for a scaling constant *e*. The rationale of the urn model is to emulate, by the first draw, the frequency that an agent of a certain opinion happens to persuade another agent. The second draw models the average success rate of the persuasion based on the current global state. Thus, the urn model has no explicit concept of group sizes as Galam's model and only implicitly assumes a minimal setting of a bilateral meeting. Also spatial correlations of agents are not incorporated explicitly but can be represented by the probability of positive feedback *P*_fb_(*s*).

**Figure 2:**
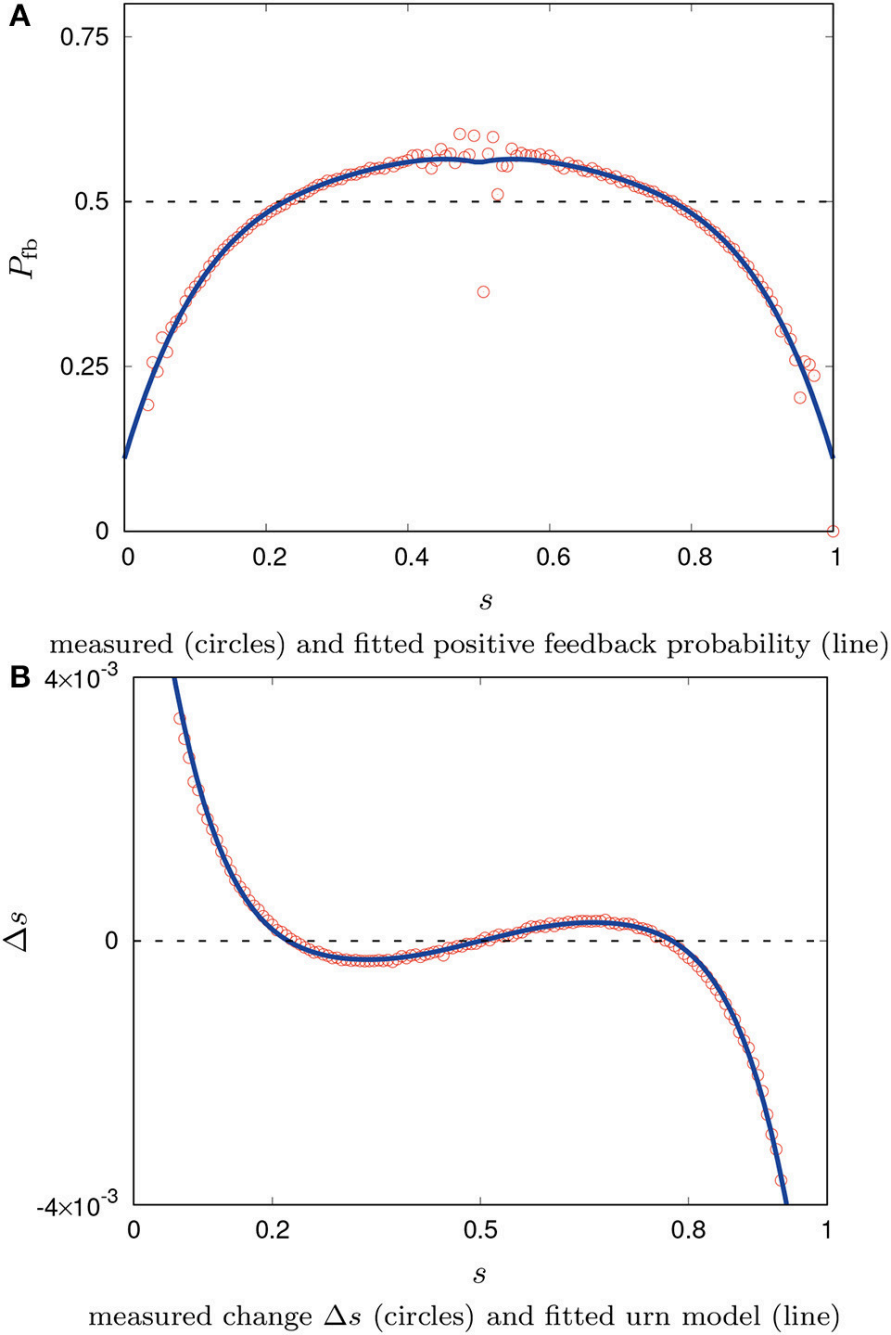
Measured positive feedback with fitted polynomial for the urn model and system dynamics Δ*s* compared to measurements of the agent-based model.

Following Hamann (2013), the probability of positive feedback can be measured in the simulation based on observations of opinion revisions

$$\begin{array}{rcl} P_{\text{fb}}\left(s\right) & = & \frac{\frac{r_{b}\left(s\right)}{r_{b}\left(s\right)+r_{a}\left(s\right)}-1+s}{2s-1},\text{for}s\neq 0.5, \end{array}$$

(8) $$\begin{array}{r} \;\min\left(s,1-s\right)\;\leq \frac{r_{b}\left(s\right)}{r_{b}\left(s\right)+r_{a}\left(s\right)}\leq \max\left(s,1-s\right), \end{array}$$

for *r*_*b*_(*s*) is the absolute number of observed individual decision revisions from opinion *A* to *B* over any given period and *r*_*a*_(*s*) denotes revisions from *B* to *A*. The measured function *P*_fb_(*s*) is fitted by a polynomial of 4th degree^6^ which is set mirror-symmetrical in *s* = 0.5. The result is shown in Figure 2A. Based on this empirically obtained function *P*_fb_(*s*) the dynamics of the system is then defined by Equation(7). A comparison to data from simulations is shown in Figure 2B which shows a very good fit^7^.

## 5. Discussion and conclusion

We have reported two approaches to overcome the limitations of the well-mixed assumption in models of opinion dynamics. Both approaches are a combination of mathematical modeling and empirically obtained parameters.

The 2-state Galam opinion dynamics model with contrarians has systematic errors for extreme values of *s*, see Figure 1D. We chose to interpret the effect of spatial correlations as a contrarian effect. Hence, we used the main empirical element of Galam's model, parameter *a* specifying the fraction of contrarians, but the model still suffers from the simplifying assumption of well-mixed particles. The errors could also not be overcome by simple extensions of fitted *s*^loc^-functions (data not shown). Despite this shortcoming, we gain a valid insight. A spatial correlation can be a local group of particles that share a similar opinion. However, they can be contrarian to the global majority. While this local group of particles acts according to properly defined decision rules, its global effect is that they oppose the majority as if they would defect the system in the way Galam's contrarians do.

In the case of the urn model, a very good fit to the simulation data was obtained using the probability of positive feedback that is measured following Equation (8). The urn model has a comprehensive empirical element [*P*_fb_(*s*)] and is still simple, concise, and achieves high accuracy. Measuring the positive feedback probability seems to comprise the averaged influence of correlations in the agents' spatial distribution. The urn model, hence, could be used to predict the long-term behavior of the collective system. The gained insight from this second modeling approach is that spatial correlations may be difficult to measure but they can be captured with a concise global modeling approach.

In addition, the knowledge about Δ*s* can be used as a novel tool in multiple ways to model opinion dynamics in mobile agents. For example, one can macroscopically model the system dynamics as a Markov chain (Valentini et al., 2014, 2017) or by Langevin and Fokker–Planck equations (Carlen et al., 2013; Hamann, 2013) which allows for good predictions without modeling spatial distributions explicitly. Features such as the steady state of the probability density function of the global opinion state *s* or the mean first passage time (i.e., the switching time between two states of consensus) can be predicted with such models (Yates et al., 2009). Another interesting aspect is to apply the concept of the local opinion state $s_{A,B}^{\text{loc}}\left(s\right)$ within an agent to find an accurate estimate of the global state based on local sampling. One faces a kind of a bootstrapping problem then, because the agent only has a local sample instead of the actual global state. However, it seems feasible that systematic spatial correlations could be reduced by such a modeling approach. This would be useful especially in swarm robotics.

## Author contributions

The author confirms being the sole contributor of this work and approved it for publication.

### Conflict of interest statement

The author declares that the research was conducted in the absence of any commercial or financial relationships that could be construed as a potential conflict of interest.

## Footnotes

^1^Note that the distance *r* = 0.01 is not always enforced because of the particles' high velocity of 0.01 per time step. Distances between particles below *r* = 0.01 do occur. Once such an event is detected particles turn away from each other.

^2^Choice of five is arbitrary, while odd numbers are preferred to avoid tie-breaking methods; the agent does not change its opinion until five encounters have occurred; if there are more neighbors, a random subset is chosen to keep the limit of five encounters.

^3^$s_{1}^{*}=0$ and $s_{2}^{*}=1$ are stable fixed points because in these states all particles have the same opinion and without noise these system states are absorbing (i.e., once reached the system cannot leave them anymore). Also note the instable fixed point *s*_3_ = 0.5.

^4^Summed squared error: 4.741 × 10^−3^.

^5^This equation is easily obtained by basic probability theory considering all four cases of drawing an agent of either color followed by either positive or negative feedback.

^6^polynomial $f\left(x\right)=c_{3}x^{4}+c_{4}x^{3}+c_{5}x^{2}+c_{6}x+c_{7}$ with 0 ≤ *f*(*x*) ≤ 1 to model a probability, *c*_3_ = −21.3144, *c*_4_ = 29.7651, *c*_5_ = −16.1583, *c*_6_ = 4.2788, *c*_7_ = 0.0720295.

^7^Summed squared error: 3.715 × 10^−7^.
